# Preparation and Assembly of the Axial Invasion Chamber for Live-Cell Invadopodia Imaging

**DOI:** 10.21769/BioProtoc.5658

**Published:** 2026-04-20

**Authors:** Mark Garewal, Kenneth A. Myers

**Affiliations:** Department of Biology, Saint Joseph’s University, Philadelphia, PA, USA

**Keywords:** Invadopodia, Live-cell imaging, Confocal fluorescence microscopy, Cell invasion, Matrix degradation, Actin-rich protrusions

## Abstract

Metastasis is initiated by cell invasion of the basement membrane, facilitating cell migration and colonization at a secondary tumor site. Cancer cells remodel the cytoskeleton to form ventral protrusions, termed invadopodia, that traffic and deliver matrix metalloproteases to degrade the extracellular matrix. Traditional efforts have utilized immunolabeling to measure protein localization within invadopodia, an approach limited by reduced temporal resolution, logistical challenges in orienting invadopodia within the focal plane of the objective lens, and impaired ability to reconstitute physiological conditions. Here, we describe a protocol for constructing and utilizing the axial invasion chamber (AIC) to perform live-cell 3D visualization of mature elongating invadopodia under physiological conditions. The AIC is simple to build, using standard 35 mm glass-bottom dishes that suit most microscope stage holders. A polyester membrane is used to uniformly orient and promote invadopodia formation and restrict cell migration. The AIC extracellular matrix is composed of readily available reagents that have been optimized to facilitate cell adhesion and invadopodia maturation. Critical advances of the AIC include imaging and measurements of protein localization without immunolabeling, imaging of live cell invadopodia using conventional inverted microscopes, and production of a fully operational apparatus within 28 h from initial assembly. While the protocol has been used for live-cell invadopodia protein localization and structure, it provides an opportunity to interchange components of the polyester membrane and/or the extracellular matrix to optimize the device for a variety of different cell types and cell invasion studies.

Key features

• Enables high-resolution live-cell invadopodia imaging along the axial plane and visualization of protein localization and length of protrusion.

• Live-cell imaging with transient transfection of fluorescent proteins and interchangeable components to study various aspects of cell invasion and migration.

## Graphical overview


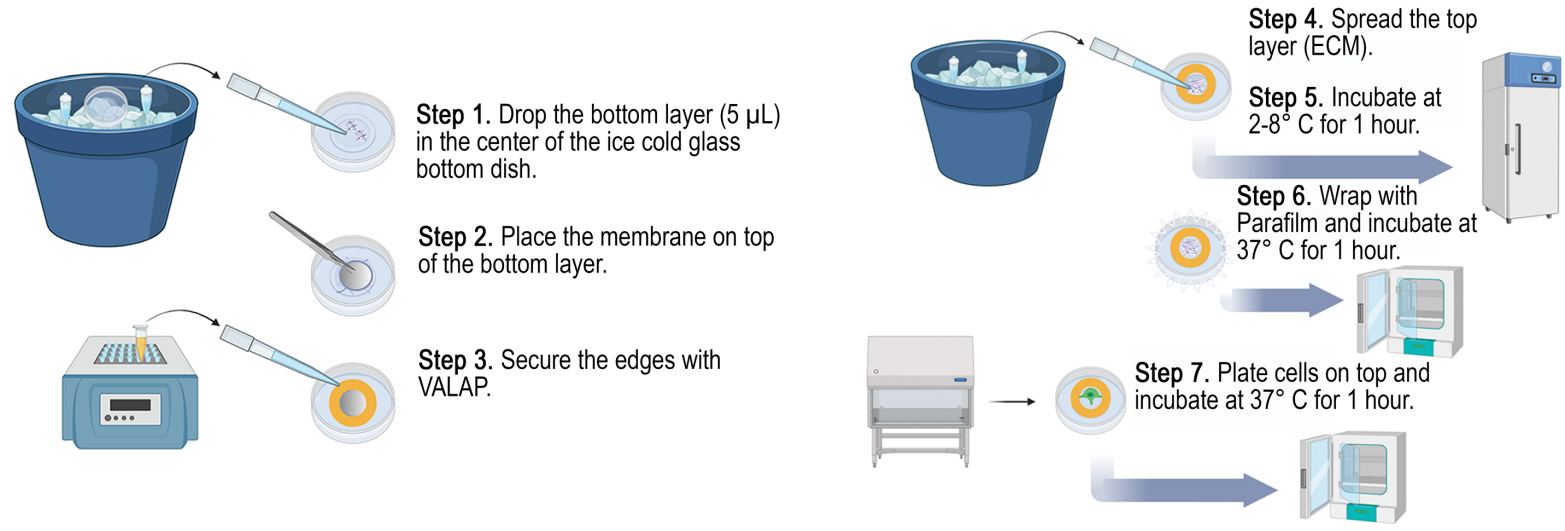


**Schematic of axial invasion chamber assembly.** Steps to build the axial invasion chamber (AIC). Steps 1–3: building the base layers; steps 4–6: constructing the top layers and polymerizing both layers (top and bottom) of the extracellular matrix (ECM).

## Background

Hallmarks of cancer that include epithelial–mesenchymal transition (EMT), increased cell motility, immune system evasion, and unlimited cell proliferation, among others, contribute to the cancer cell phenotype for sustained tumor progression [1]. Notably, all hallmarks are shared between malignant and benign tumors, except for cell invasion and metastasis, which is considered highly critical and the most elusive [2]. Despite these findings, clinical research targeting cancer cell metastasis and secondary lesions remains far less prevalent than studies aimed at inhibiting the primary tumor cell hallmarks. Furthermore, tumor cell dissemination and migration to another colonizing site is a major cause of death in cancer patients [3]. Thus, it is imperative to investigate the molecular mechanisms of metastasis to enable the development of therapies with greater impact on patient survival.

The process of establishing secondary tumor sites is known as the metastatic cascade, being initiated by cell invasion and degradation of the extracellular matrix (ECM) beyond the basement membrane [3–5]. Cancer cells remodel the cytoskeleton to form an actin-rich ventral protrusion that facilitates trafficking and delivery of matrix metalloproteases (MMPs), termed invadopodia [6–9]. Proteomic analyses have identified more than 70 proteins that localize to invadopodia; however, the functional roles of many of these components remain largely unknown [10]. Thus, there is a clear gap and need for a research tool to study the dynamic behaviors and functional roles of these proteins during cancer cell invasion. Previous research methods to study protein localization within invadopodia used cells cultured on a thin layer of fluorescent matrix to search for areas of ECM degradation and to co-localize fluorescent biomarkers [11,12], while studies including tumor spheroid imaging and observing cells embedded within matrices have also been attempted [13–16]. However, prior investigations are limited by fixed immunolabeling, inconsistent orientations of cancer cell protrusions that create a logistical challenge to image cells within the focal plane of the objective, and cell culture systems using atypical physiological conditions.

**Figure 1.**
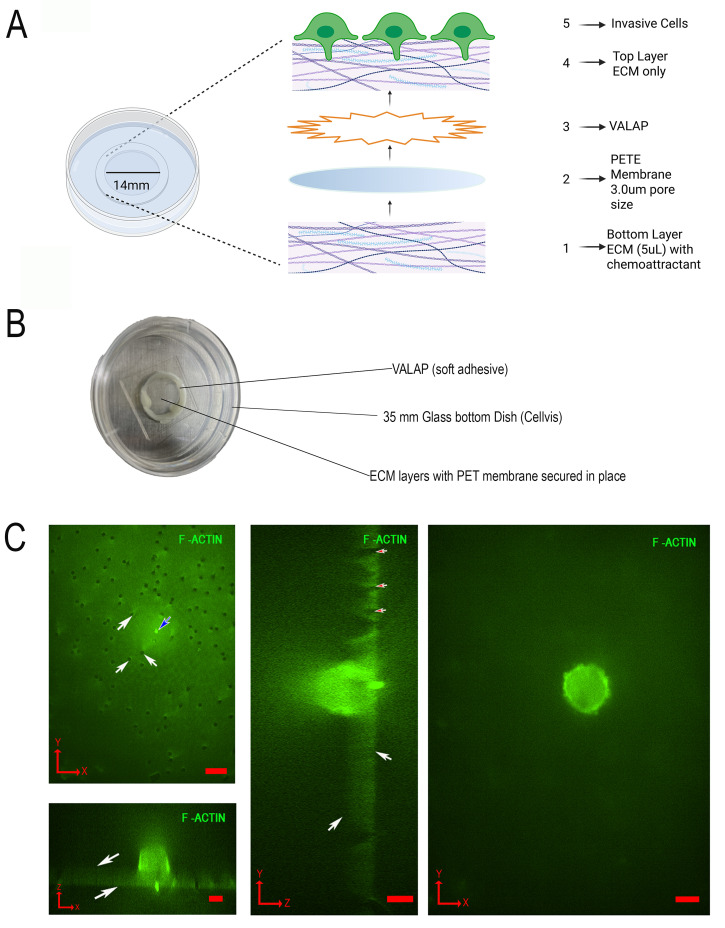
Design and construction of the axial invasion chamber (AIC). (A) Zoomed out view of all layers constructed during assembly from bottom to top (numbered 1–5). (B) Picture of assembled AIC. (C) Representative coordinate plane images of an invading MDA-MB-231 cell cultured in the AIC. The cell is expressing GFP-F-Actin, and images were acquired by spinning disk confocal fluorescence microscopy with 60× magnification. (Top left) XY image of the bottom of the PETE membrane, showing invadopodia protrusion through the 3.0 μm pore (blue arrow) and non-protruding pores (white arrows). (Bottom left) XZ image showing the top and bottom of the PETE membrane (white arrows). (Middle) YZ image showing pores (red arrows) and the top and bottom of the membrane (white arrows). (Right) XY image of a cell viewed from the top of the membrane. All scale bars, 10 μm. Red arrows in the bottom left corners indicate the axis orientation as pictured (X, Y, and Z). Panels A and B, adapted from Garewal et al. [17] MBoC, licensed from CC BY 4.0.

Here, we created an axial invasion chamber (AIC) that is capable of live-cell imaging with transient expression of proteins, a consistent orientation for invadopodia, and optimized for physiological conditions of the ECM (Figure 1). The AIC consists of a bottom chemoattractant ECM layer, with an adhesive-secured transparent 3.0 μm pore size membrane placed on top, followed by coating and polymerization of the membrane with ECM. Cells are then cultured on top of the coated membrane for 24 h to invade, followed by imaging. Limitations of the AIC consist of decreased resolution in the Z-plane due to fluorescent light scattering through the ECM, along with rapid photobleaching, a limited cell culture area defined by the bottom well dish diameter, and the required use of a long working distance objective lens. The AIC provides experimental design opportunities via interchangeable components, including various membrane pore sizes and ECM compositions, useful for cell invasion studies, such as blood–brain barrier and invasion-mediated whole cell migration.

## Materials and reagents

**Biological materials**

1. Human breast cancer metastatic cell line (MDA-MB-231; human origin; catalog number: ATCC HTB-26)

**Reagents**

1. Dulbecco’s modified Eagle’s medium (DMEM), sterile, high glucose with L-glutamine, without phenol red, sodium pyruvate, liquid (Cytiva, catalog number: SH30284.01)

2. Sodium pyruvate solution, sterile-filtered, 100 mM (Cytiva, catalog number: SH30239.01)

3. Fetal bovine serum, 500 mL, regular, USDA-approved origin (heat inactivated) (Corning, catalog number: 35-011-CV)

4. BioReagent penicillin-streptomycin, sterile, 100×, 10,000 U/mL penicillin, 10 mg/mL streptomycin, solution stabilized, suitable for cell culture, liquid (Sigma-Aldrich, catalog number: P4333-100ML)

5. Lipofectamine 3000 transfection reagent (Thermo Fisher Scientific, catalog number: L3000008)

6. Trypsin EDTA 1× (Corning, catalog number: 25-053-Cl)

7. Geltrex^TM^ LDEV-free reduced growth factor basement membrane matrix (Thermo Fisher Scientific, catalog number: A1413202)

8. BioReagent Laminin, 1–2 mg/mL, sterile, from Engelbreth–Holm–Swarm murine sarcoma basement membrane, suitable for cell culture (Sigma-Aldrich, catalog number: L2020)

9. hEGF, EGF, recombinant, expressed in *E. coli*, lyophilized powder, suitable for cell culture (Sigma-Aldrich, catalog number: E9644)

10. Petroleum jelly (Medline, catalog number: CUR0053457)

11. Paraffin (Sigma-Aldrich, catalog number: 708860)

12. Lanolin (TagetMol, catalog number: T3238)

13. Dulbecco's phosphate-buffered saline, modified (DPBS), w/o calcium chloride and magnesium chloride, filter-sterilized (Sigma-Aldrich, catalog number: D8537)

14. HEPES solution,1 M, pH 7.0–7.6, filter-sterilized (Sigma-Aldrich, catalog number: H0887-20M)

**Solutions**

1. Cell culture media mix (see Recipes)

2. ECM bottom layer mix 20× stock solution (see Recipes)

3. ECM top layer mix 6× stock solution (see Recipes)

4. VALAP 1:1:1 mix (see Recipes)

**Recipes**

**1. Cell culture media mix**

**Table:** 

Reagent	Final concentration	Quantity or volume
DMEM	88.5%	500 mL
Sodium pyruvate	0.88%	5 mL
Fetal bovine serum	8.85%	50 mL
Penicillin-streptomycin	1.77%	10 mL
Total	100%	565 mL

Store at 2–8 °C for up to 1 month.

**2. ECM bottom layer mix 20× stock solution**

**Table:** 

Reagent	Final concentration	Quantity or volume
Geltrex:Laminin 1:1 mix	10%	10 μL
hEGF	9%	9 μL
Cell culture media mix	8%	8 μL
Fetal bovine serum	73%	73 μL
Total	100%	100 μL

 Aliquot and store at -20 °C for up to 6 months.

**3. ECM top layer mix 6**× **stock solution**

**Table:** 

Reagent	Final concentration	Quantity or volume
Geltrex:Laminin 1:1 mix	10%	30 μL
Cell culture media mix	90%	270 μL
Total	100%	300 μL

Aliquot in 300 μL and store at -20 °C for up to 6 months.

**4. VALAP 1:1:1 mix**

**Table:** 

Reagent	Final concentration	Quantity or volume
Petroleum jelly	33.3%	30 g
Paraffin	33.3%	30 g
Lanolin	33.3%	30 g
Total	~100%	90 g

Heat on a hot plate until in a liquid state. Aliquot in 1.5 mL Eppendorf tubes to place in the biosafety cabinet (BSC) and store at room temperature for up to 2 years.

**Laboratory supplies**

1. 35 mm glass-bottom dishes (Cellvis, catalog number: D35-14-1.5-N)

2. Polyester track etch (PETE) membrane filters, transparent, 3.0 µm, 12 µm thickness, 6 × 10^5^ pores/cm^2^, 13 mm, 100/pack (Steriltech, catalog number: 1300025)

3. 225 cm^2^ polystyrene tissue culture treated flasks, sterile, vent cap, canted neck (Celltreat, catalog number: 229371)

4. 12-well polystyrene tissue culture treated multiple well plates, sterile, individually wrapped, with lid (Fablab, catalog number: FL7111)

5. 5 mL serological pipette, individual plastic/plastic wrapper packed in bags, sterile (Celltreat, catalog number: 229205A)

6. 10 mL polystyrene serological pipettes, sterile, individually paper/plastic wrapped, color coded orange (Celltreat, catalog number: 667210B)

7. Corning^®^ 1–200 μL universal fit racked pipette tips, natural, sterile (Corning, catalog number: 4864)

8. Corning^®^ 100–1,000 μL universal fit racked pipette tips, blue, sterile (Corning, catalog number: 9032)

9. Razor blades

10. 15 mL polypropylene centrifuge tubes, sterile, bulk bag (Celltreat, catalog number: 667015B)

11. Eppendorf safe-lock tubes, 1.5 mL, colorless (Eppendorf, catalog number: 022363204)

14. Pyrex^®^ Petri dish bottom only, O.D. × H 100 mm × 20 mm

15. Hemacytometer

16. Parafilm

## Equipment

1. Biosafety cabinet (BSC)

2. TiE microscope with stage, laser, camera, computer, etc. (Nikon, catalog number: 12831)

3. Tweezers

4. Cell culture incubator at 37 °C, 5% CO_2_, 90% relative humidity (Thermo Fisher Scientific, catalog number: 51030401)

5. Water bath 37 °C (Thermo Fisher Scientific, catalog number: FSGPD20)

6. Benchtop centrifuge for 15 mL conical tubes (Benchmark Scientific, catalog number: WBB3113837)

7. Freezer -20 °C

8. Refrigerator 2–8 °C

9. Large diameter >10” ice container

10. Hotplate (Thermo Fisher Scientific, catalog number: 1152049H)

11. Digital heat block with holes to accommodate the diameter of 1.5 mL Eppendorf tubes (VWR, catalog number: 15259-050)

## Software and datasets

1. NIS elements version 4.3; license needed

## Procedure

**A. Twenty-four-hour advanced cell preparation**

1. Culture MDA-MB-231 cells in tissue culture–treated flasks from an initial plating of ~500,000 cells until a confluency of 80%–90% has been reached with the cell culture media mix. Remove and replace media every 2 days. Warm the cell culture media to 37 °C before working with the cells.

2. Once 80%–90% confluency has been reached, aspirate cell culture media and rinse cells twice with 5 mL of 1× PBS. Aspirate PBS from the flask and add 1 mL of 0.05% trypsin. Incubate the trypsin and cells in the flask at 37 °C for no more than 2 min, followed by immediate addition of 10 mL of cell culture. Pipette vigorously to remove cells from the bottom of the flask, transfer cells to a 15 mL conical tube, and centrifuge at 300–500× *g* for 3 min. Aspirate trypsin/media mixture and resuspend the cell pellet in 1 mL of fresh media. Pipette 10 μL of the resuspension into the groove of a hemacytometer and place the hemacytometer-specific coverslip on top. Using a microscope at 10×, count the number of cells in five squares, including the middle square. Record the total cells from all squares, divide by 5 (total number of squares), and multiply by 10,000 (correction factor) to determine cells per milliliter. Use the cells/mL determined from the cell count to aliquot appropriate volumes to use in AIC plating.

3. In a 12-well plate, plate ~200,000 cells in 2 mL of cell culture media for each well, as needed for the number of AICs planned, with two extra wells to account for possible errors. Approximately 150,000–200,000 cells are needed for final plating per AIC.

4. Two hours prior to assembling the AIC (4 h total transfection time), transfect cells directly in the 12-well plate with cDNA and Lipofectamine 3000 at a 1:4 ratio, as per the manufacturer’s recommendations. See MDA-MB-231 or appropriate cell line transfection instructions for Lipofectamine. Incubate the cells in the 12-well plate at 37 °C for 4 h.

**B. Assembly of the AIC**

1. Fill a large ice container with sterile ice (DI-water derived), leaving 2 inches of space from the top, and place it in the BSC. Ensure the ice is packed and as smooth as possible.

2. Thaw aliquots of ECM bottom and top layer mixes (see Recipes) in the same large ice container in the BSC. Place aliquot Eppendorf tubes toward the sides of the ice container.

**Caution:** Spray all items with 70% ethanol before placing them in the BSC, as all items need to be sanitized.

3. While the ECM top and bottom layer mix aliquots are thawing, turn on the heat block, set it to 121 °C (in the BSC), and heat two VALAP 1.5 mL Eppendorf tubes for ~30 min.

4. Place a single top or bottom piece of a Pyrex large Petri dish flat on the large ice container in the BSC, ensuring that the entire dish is in contact with the ice.

5. Place 2 (or up to 6) 35 mm glass-bottom dishes on the Pyrex Petri dish cover and let them cool for ~20 min.

*Note: All subsequent references to placing glass-bottom dishes on ice mean placing them in the Pyrex Petri dish cover that is on ice.*

**Caution:** Depending on the humidity in your cell culture room, the glass-bottom dishes may build too much condensation if left on ice for too long. The condensation will affect your bottom layer mix by adding extra volume and modifying ECM concentration.

6. Cut a 10 μL pipette tip with a razor blade, approximately 1/8” inch from the tip.

**Critical:** Pipette tips should always be cut when dispensing the Geltrex:Laminin mix, as the ECM is viscous.

7. Dispense 5 μL of the bottom layer mix as a single drop in the center of the glass-bottom dish and place immediately back on ice. Keep the dish on ice whenever possible. Repeat for all dishes and let them sit on ice for at least 5 min.

8. Use tweezers to remove filter membranes from the package box, close the box, and place the membranes on top of the box, with the membrane edge slightly placed off the box for ease of removal from the filter paper.

**Caution:** The membranes are very thin, and airflow can cause the membranes to scatter around the BSC. Handle them carefully from the edges with tweezers.

9. Carefully and slowly place the membrane centered on top of the aliquoted bottom layer mix drop.

**Critical:** Do not move the membrane once it is placed on top of the aliquot drop. This requires practice, as handling the thin membrane with tweezers and airflow within the BSC can be quite challenging.

10. Cut another 10 μL pipette tip 1/8” inch from the tip and quickly dispense the hot VALAP (~121 °C) into the tip. Immediately drop the VALAP around the edge of the membrane to seal the membrane and the bottom layer mix, and to the side wall of the well.

**Caution:** Do not spread the VALAP with the pipette tip; continually drop it in place around the edge of the membrane and hold the dish at a 45° or greater angle to help the VALAP drop into place around the edge.

11. Once the membrane has been secured with VALAP, place the glass-bottom dish on ice for at least 10 min.

**Pause point:** At this stage, you can pause the assembly for as long as the dish remains on ice.

12. After a 10-min incubation on ice, apply and gently spread 6 μL of the top layer mix with a cut 10 μL pipette tip (see step B6). Keep the dish on ice whenever possible.

13. Take the Pyrex Petri dish with assembled AICs and place it in a refrigerator at 2–8 °C for 1 h to allow the bottom layer mix ECM to stabilize and the top layer mix to spread evenly throughout the PETE membrane.

**Pause point:** AICs can remain in the refrigerator at 2–8 °C for more than 1 h, but do not exceed 3 h, as the ECM may dehydrate.

14. While AICs are incubating in the refrigerator at 2–8 °C, cut Parafilm into 2 × 6-inch strips, for a total of 3 strips for each AIC.

15. After the 1-h incubation, wrap the AICs with the lid on around the circular edge with 3 Parafilm cut strips to triple wrap the entire perimeter of the AIC.

**Caution**: Ensure the entire perimeter is covered **at least 3 times**, or the ECM will dry out in the incubator.

16. Incubate the AICs in the Pyrex cover dish for at least 30 min in a 37 °C incubator.

**C. Cell preparation for AIC plating**

1. While the AICs are incubating, remove the transfected MDA-MB-231 cells (see step A4) from their dishes by aspiration of the cell culture media, followed by two rinses with 1 mL of PBS per well. Aspirate the PBS, add 500 μL of 0.05% trypsin per well, and incubate the 12-well plate at 37 °C for no longer than 2 min. Add 2 mL of cell culture media, followed by vigorous pipetting to remove all cells from the bottom of each well, and place in 15 mL conical tubes.

2. Centrifuge the 15 mL conical tubes and resuspend in 2 mL of cell culture media to count with a hemacytometer.

3. Plate ~200,000 cells in 200 μL of final cell culture media on AICs and incubate for 24 h.

4. Thirty minutes prior to imaging, add 35 mM of HEPES to 2 mL of cell culture media.

5. Prepare the microscope for imaging by heating the stage with an air blower or enclosure that is calibrated to provide a temperature close to 37 °C and a 40×–60× magnification objective lens with at least a 310 μm working distance. The objective’s working distance is required to image cells in focus from the top of the cell to the distal tip of invadopodia. A working distance lower than 310 μm will not allow a full z-stack image from the top of the cell to the invadopodia bottom.

## Data analysis

**Image acquisition and data analysis**

Here, we present a method to acquire Z-stack images and perform computational measurements of invadopodia depth using cDNA co-expression of a membrane marker (CAAX), combined with fluorescently labeled proteins within invadopodia. The methodology described below is specific to Nikon Imaging Software (NIS) Elements software but can be adapted to other imaging software with the same measurement principles. Microscope system automation was controlled with NIS, and Z-stack images of fluorescently labeled constructs were taken using laser illumination (405, 488, and 561 nm) provided by a Nikon LUN-F XL 405/488/561/640 nm laser combiner (50/60/50/40 mW) that was shuttered with electronic shutters and directed to the confocal scan-head via a FC-PC8 output fiber (Nikon) with exposure times of 500 ms. After acquisition, general image processing involved optimization of image brightness and contrast using the Nikon Elements software package.

**Figure 2.**
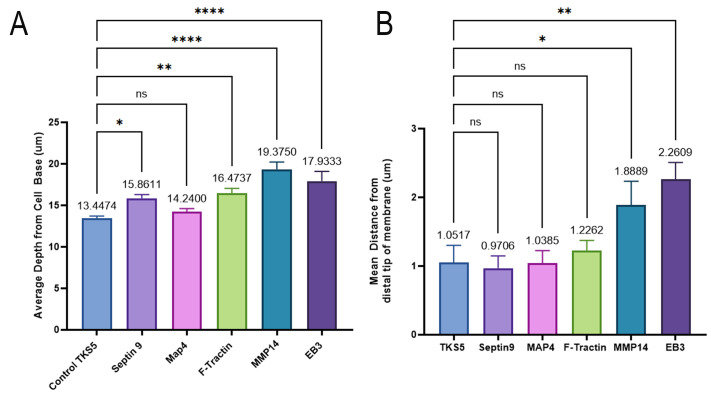
Data analysis of invadopodia measurements with the axial invasion chamber (AIC). (A) Depth of expressed proteins from the cell base. (B) Mean distance from the distal tip of the membrane of expressed proteins using the CAAX membrane marker. GraphPad Prism v10 was used to generate graphs and for statistical analysis (one-way ANOVA); data are mean + standard error of the mean (SEM). *p < 0.05, **p < 0.005; ***p < 0.0005; ****p < 0.00001. ns, non-significant.

1. Open NIS elements. In ND Acquisition mode, set exposure time to 500 ms (or suitable exposure for your imaging system and laser power), set laser power to 25%, set Z-stack range to 90 μm top to bottom, and click on the *symmetric range home* button. Use epifluorescence light with the appropriate excitation filter to locate a fluorescent cell in the eyepiece of your microscope. Adjust the focal plane slightly to locate the bottom of the membrane. Membrane pores are visible by auto-fluorescence, and protrusions are bright and easy to visualize at this Z-position of the membrane. Once a cell has been identified, quickly turn off the epifluorescence light to avoid photobleaching. Name the file in the appropriate folder and click *run now* to image the z-stack file.

2. To open the saved file and perform measurements, click *Open File > Auto scale button on top of z-stack file window > Slices view button*.

3. A new window will open. Slowly move the slider to the left (toward the top of the cell in the z-stack) until the membrane pores are in focus near the protrusion. Note this z-position in a spreadsheet (paper or electronic), as this is the bottom of the PETE membrane.

4. Now, measure the maximum depth of the expressed protein or the plasma membrane (CAAX marker) by moving the slider to the right until the fluorescent signal is no longer in focus. This can be easily visualized by the XZ and YZ windows in the *Slices view* window of NIS Elements. Note this location from the z-position bottom slider. Steps 2–4 can be repeated for additional wavelengths, if desired.

5. Using the spreadsheet, create columns with the following titles for cells A–H and enter data as follows: A) Cell number (enter each individual cell number or unique identifier for each z-stack set of images), B) fluorescently labeled constructs (enter the construct information, i.e., GFP-Actin/RFP-CAAX), C) PETE membrane distance measurement (enter the position from step 3 above in this section), D) maximum distance from cell bottom for GFP-Actin (enter the position from step 4), E) maximum distance from cell bottom for CAAX (plasma membrane marker), F) GFP-Actin distance (enter formula = (D3-C3)-12; the formula equates the distance of GFP-Actin from the cell base to the distal tip of invadopodia), G) RFP-CAAX distance (enter formula = (E3-C3)-12; the formula equates the distance of the plasma membrane from the cell base), and F) delta [enter the formula = G3-F3; the formula equates the difference of the fluorescently labeled protein of GFP-Actin from the plasma membrane (CAAX)]. Figure 2 represents data collected using this method for various cytoskeletal-related proteins.

## Validation of protocol

This protocol has been used and validated in the following research article:

- Garewal et al. [17]. A structured framework of cytoskeletal proteins and noncentrosomal microtubules promotes the initiation and elongation of invadopodia. *Mol Biol Cell* (Figures 2, 3, and 4).
  Development of the AIC first required the selection of a PETE membrane that was transparent and amenable to cell culture and had the appropriate pore size amenable to high-resolution imaging. Further development of the bottom-layer composition required a robust chemoattractant diffusion from the bottom layer through the PETE membrane into the top layer. In addition, the top ECM layer required optimization to induce cell invasion. Lastly, a fabrication process was required to construct the AIC, enabling uniform ECM distribution, securely sealing the PETE membrane to isolate the bottom layer, and allowing controlled polymerization of both the top and bottom ECM layers to provide an environment suitable for cell invasion.
  Chemoattractant studies were conducted in invasion chambers (ICs) and compared to conventional Boyden chamber (BC) assays used in other cell invasion studies [18,19]. Various compositions of the bottom layer were used to study the diffusion through the BC PETE membrane and were compared to the IC. Diffusion rates were determined by total protein measurement from the top layer over 24 h at 0, 4, and 24 h. Data was plotted, and simple regression lines were compiled with line equations and slopes to indicate the rate of diffusion. IC compared to BC diffusion rates were significantly reduced (Figure 3A). Slower rates suggest a sustained, longer chemoattractant gradient as robust diffusion with the IC, validating the fabrication process. Optimization of the top ECM layer composition was achieved using Geltrex, an ECM known to induce MDA-MB-231 cell invasion at ~1–2 mg/mL [20]. Various concentrations of the top ECM layer were tested for total protein concentration and effects on cell invasion (Figure 3B, C). These validation experiments revealed that the 1:10 Geltrex:Laminin mixture was the most effective at achieving protein concentrations closest to the target range of ~1–2 mg/mL (1.79 mg/mL; Figure 3B), while measurements of cell invasion revealed that the 1:10 Geltrex:Laminin combination produced the greatest number of cell invasion events (5.4 cells per AIC; Figure 3C). Given the outcomes of these validation experiments, the 1:10 Geltrex:Laminin top ECM layer was selected to be used with the AIC (Figure 3B).
  **Figure 3.**
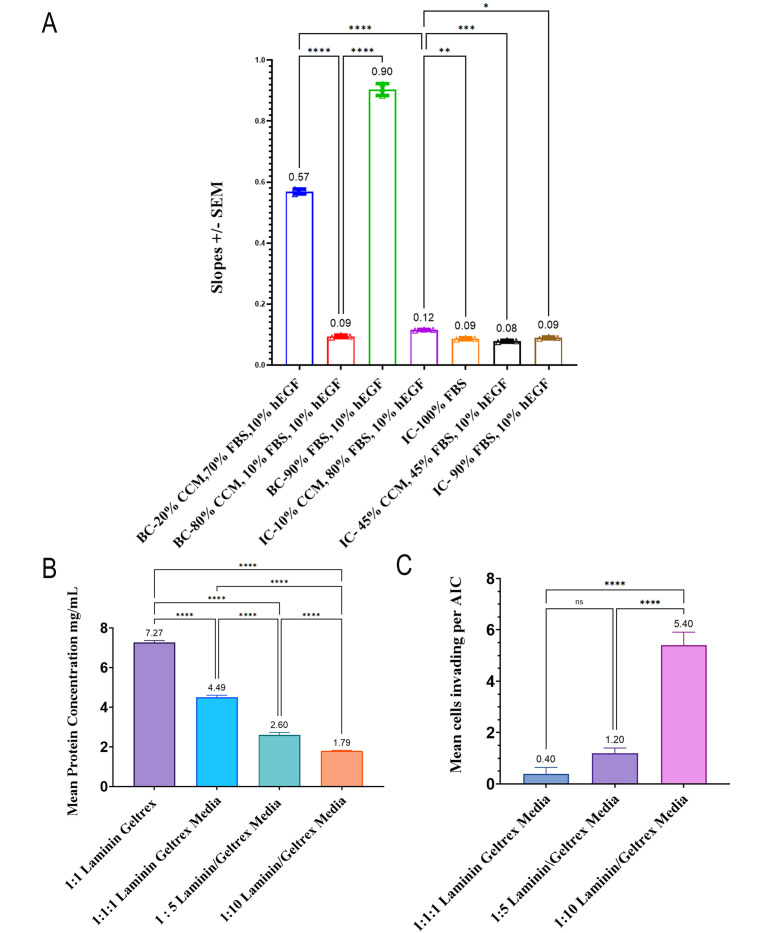
Development of the axial invasion chamber (AIC) showed robust chemoattractant diffusion from the extracellular matrix (ECM) bottom and optimal top layers to induce cell invasion–mediated invadopodia. (A) Total protein slopes for different bottom layer compositions with abbreviations as follows: Boyden chamber (BC), invasion chamber (IC), cell culture media (CCM), fetal bovine serum (FBS), and human epidermal growth factor (hEGF). (B) Mean protein concentration with different dilutions of the ECM top layer mix. (C) Mean cells invading the AIC dish with different top layer ECM dilution conditions. Error bars are the standard error of the mean (SEM). Statistical analysis: ANOVA. *p < 0.05; **p < 0.005; ***p < 0.0005; ****p < 0.0001. ns, non-significant.

## General notes and troubleshooting

**General notes**

1. Ensure the AIC is placed in the Pyrex petri dish cover plate ensuring full contact with the ice layer at all times prior to incubation at 37 °C.

2. Use fresh ECM aliquots by limiting freeze/thaw cycles to a maximum of 3.

3. Ensure the Geltrex, laminin, and hEGF are not older than 6 months. Fresh ECM is required to induce cell invasion.

4. Limit cell passage to no more than 10 to promote robust cell invasion.

**Troubleshooting**

1. Cells do not invade but express fluorescently tagged proteins: Ensure that cell confluency is >80% on the AIC and passage is not higher than 10.

2. ECM dries during the 37 °C incubation step: Wrap the Parafilm tightly around the glass-bottom dish in at least three layers. Two layers are not sufficient and can result in ECM dehydration.

3. Membrane floated up during the 24-h incubation: Ensure that an adequate amount of VALAP is applied around the edge of the membrane. Ensure that the VALAP dries for at least 10 min on ice before adding the top ECM layer.
